# Ertapenem and Faropenem against *Mycobacterium tuberculosis*: in vitro testing and comparison by macro and microdilution

**DOI:** 10.1186/s12866-020-01954-w

**Published:** 2020-08-31

**Authors:** Ximena Gonzalo, Giovanni Satta, Julio Ortiz Canseco, Timothy D. McHugh, Francis Drobniewski

**Affiliations:** 1 Department of Infectious Diseases, Section Adult Infectious Diseases, Imperial College London, 8th Floor, Commonwealth Building, Hammersmith Campus, Du Cane Rd, Shepherd’s Bush, London, W12 0HS United Kingdom; 2 Centre for Clinical Microbiology, Division of Infection & Immunity, University College London, Royal Free Campus, Rowland Hill Street, London, NW3 2QG UK; 3 Imperial College London, Department of Infectious Diseases, Section Adult Infectious Diseases, Imperial College London, 8th Floor, Commonwealth Building, Hammersmith Campus, Du Cane Rd, Shepherd’s Bush, London, W12 0HS United Kingdom

**Keywords:** Ertapenem, Faropenem, *Mycobacterium tuberculosis*, MDR-TB, XDR-TB, In-vitro, Macrodilution, Microdilution

## Abstract

**Background:**

Interest in carbapenems has been rising in the last few years due to the emergence of drug resistant tuberculosis. Ertapenem (ETP), given once a day parenteral, and faropenem (FAR), oral, have a better administration profile than meropenem (MEM), imipenem (IPM) and doripenem (DOR). The addition of amoxicillin-clavulanate (AMC) inhibits the hydrolysis by the carbapenemase present in *Mycobacterium tuberculosis* (MTB).

The aim of this study was to determine the in vitro activity of ETP and FAR against susceptible and resistant clinical MTB strains by two widely use methodologies, the BACTEC960 MGIT and microdilution.

**Results:**

19 clinical isolates with different susceptibility profiles and H37Rv were included. Minimal inhibitory concentration (MIC) testing was performed using two methods of different concentrations of ETP and FAR with and without AMC.

MIC50 was 2 and 8 for FAR with and without AMC by both methods. MIC90 was > 16 and > 8 by microdilution and MGIT respectively and did not change after AMC addition.

18/20 samples were resistant to the highest concentration of ETP, with and without AMC. Half of the samples had some susceptibility to FAR; addition of AMC further reduced the MIC level in seven isolates.

10/20 isolates showed susceptibility to FAR and the addition of AMC further reduced the MIC in 7 isolates. However, most of the MICs were near the limit of effectiveness (8 μg/mL).

Resistance to FAR was associated with resistance to MEM (*p* = 0.04) but not to resistance profiles of other drugs, including M/XDR status.

**Conclusions:**

The lack of ETP activity may be associated with its degradation, independent of carbapenemase, during incubation.

No susceptibility pattern to traditional drugs can predict susceptibility to FAR and susceptibility testing is not routinely available. PK/PD studies are needed as reaching the concentrations tested in these experiments may be challenging.

This work highlighted some of the limitations of carbapenem use. More evidence is needed to clarify their true impact in TB treatment and outcome, considering the financial burden, complications and microbiota changes associated with their use.

## Background

Carbapenems have been known to be effective against non-tuberculous mycobacteria since the early 1990s [1] and act by inhibiting the L,D-transpeptidases [2]. However, inactivation by beta-lactamases, together with a highly effective and cheaper oral drug regimen for drug susceptible tuberculosis (TB), limited their use in treating TB. With the advent of drug resistant TB, especially multi drug and extremely drug resistant (MDR/XDR) forms, interest in carbapenems has increased based on preliminary in vitro results suggesting that they may be active at concentrations achievable in vivo and reports that suggest some carbapenems successfully contribute to cure [3] particularly for bacteria that are replicating [4].

Ertapenem (ETP) and imipenem (IPM) had been reported as being the most efficient L,D transpeptidase inhibitors of *Mycobacterium tuberculosis* (MTB) [2]. Other studies showed that meropenem (MEM) was the most stable carbapenem in the presence of the chromosomally encoded *blaC* beta-lactamase [5] and that the addition of clavulanate, a beta-lactamase inhibitor, improved carbapenem activity, since this compound irreversibly inhibits the *blaC* enzyme present in MTB [6, 7]. However, laboratory data on drug efficacy and specificity are variable, and a wide range of MICs for these drugs are reported [8, 9].

For many years, amoxicillin-clavulanate (AMC) has been considered a potentially useful anti-TB drug and classified by WHO as Group D (not part of the core MDR-TB regimen) [10] but there was little data to support its use. Clavulanate is only available in the UK combined with either amoxicillin or ticarcillin, with the former being the only one available orally. The addition of AMC to MEM showed a synergistic effect against MTB strains at concentrations easily achievable in vivo [8]. Unfortunately MEM requires intravenous administration three times a day and there remains the risk of carbapenem-resistance selection [11]. Other carbapenems have a better administration profile: ETP is given intravenously once a day and faropenem (FAR) is available for oral administration [12, 13].

FAR showed good killing activity in an ex vivo model of TB, using the laboratory strains Erdman and H37Rv [14]. An additional advantage of this compound is that it is stable in the presence of the MTB *BlaC* enzyme, which means that it would remain active against those strains that become resistant to clavulanate [15]. ETP has also been shown to be active in vitro. However, testing is challenging since it degrades quickly at 37 °C [16].

Information regarding clinical use and outcomes in humans is starting to emerge, showing results suggestive of activity against MTB. The only observational study comparing imipenem/clavulanate- versus meropenem/clavulanate-containing regimens in the treatment of MDR- and XDR-TB has suggested meropenem superiority and, currently, it is the carbapenem of choice when managing multi drug resistance [17].

The aim of this study was to determine the in vitro activity of ETP and FAR against susceptible and resistant clinical MTB strains by two widely use methodologies.

The BACTEC960 MGIT system has several advantages over other methodologies, it is reliable for first and second line drug susceptibility testing, it offers automated incubation and reading and previous validation in numerous studies for the susceptibility testing of conventional and newer antimicrobials against MTB [18].

The microdilution method, on the other hand, is a flexible format that is widely used where MGIT is not available, in low throughput studies or in early stage drug development. For the same reasons and with the introduction of next generation sequencing (NGS) in the UK Mycobacterium Reference Services [19] microdilution methods have increased in prominence. This approach has been in use for several years for drug susceptibility testing (DST) in non-tuberculous Mycobacteria [19]. It is cheap and does not require additional instruments and it is usually read manually.

## Results

The results of ETP and FAR testing (with and without AMC) are shown in Table 1.

**Table 1:**
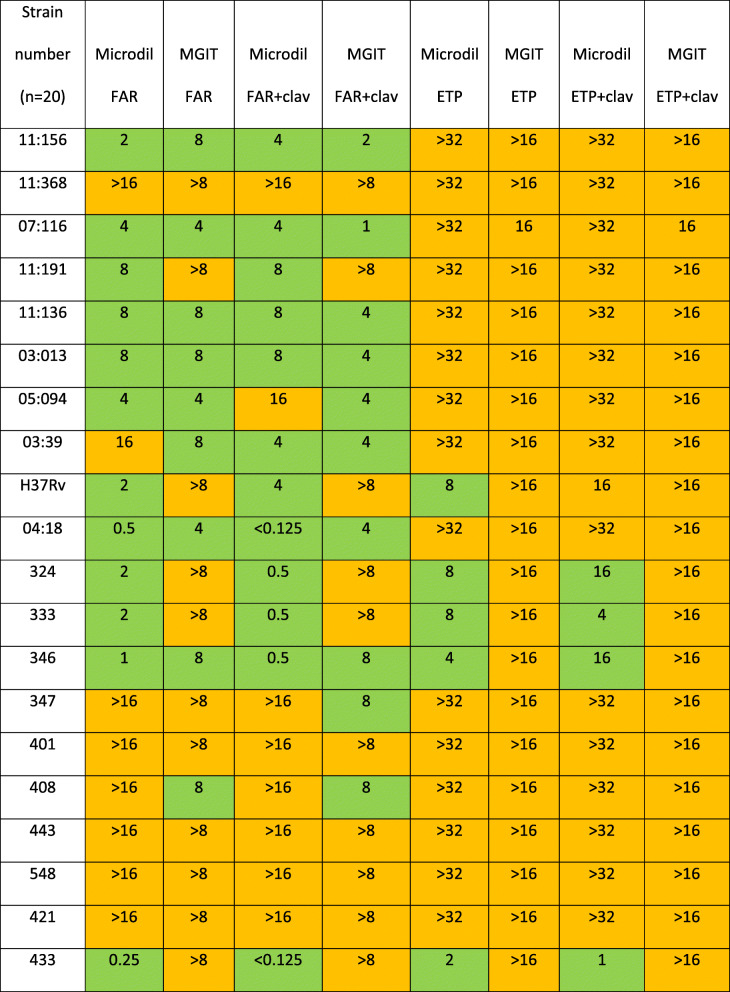
MICs of the isolates against FAR and ETP with and without AMC.

Concentrations are expressed in mg/L. Clavulanate was used at a fixed concentration of 2.5 mg/L. *FAR* Faropenem, *ETP* Ertapenem, clav Clavulanate, *microdil* Microdilution, *MGIT* Mycobacterium Growth Incubator Tube. *LJ* Lowenstein Jensen. Green shading: concentrations achievable in vivo; orange shading: concentrations above those achievable in vivo with current dosing recommendations

MIC50 was between 2 and 8 for FAR with and without clavulanate by both methods. MIC90 was > 16 and > 8 by microdilution and MGIT respectively and they did not change after the addition of clavulanic acid.

Eighteen out of twenty samples were resistant to the highest concentration of ETP tested, with and without AMC. Half of the samples tested had some degree of susceptibility to FAR and the addition of AMC further reduced the MIC level in six isolates.

Resistance or susceptibility to FAR was associated with resistance or susceptibility to meropenem (*p* = 0.04) but not to resistance profiles to other drugs, including M/XDR status.

## Discussion

There were differences in MICs obtained by the two methods used in this study. Generally, the microdilution method produced lower MICs. This may be associated with the differences in the methodologies.

ETP showed no activity against strains tested on the MGIT system (even against fully susceptible strains) and activity against 5 isolates in the microdilution model. The consistent lack of activity is likely to be an artefact associated with the reported phenomenon of ETP degradation in vitro [16]. Given the slow replication of MTB, this leads to a challenging situation in testing where the antibiotic is possibly degraded before killing or inhibiting bacterial growth. This is highlighted by the difference in the two methods where a positive readout in the MGIT would typically be at 2 to 33 days [20] whereas the microdilution method is after 7 days [21]. Some authors have also suggested the daily addition of antibiotics to this experimental set up [22] but this would hamper the evaluation of the dose tested and it would increase the risk of contamination as well as posing a repeated risk for the operator when working with M/XDR-TB. The addition of AMC did not translate into significant improvements in susceptibility. Although, ETP has been reported as useful in the treatment of TB previously as part of combination therapy, its role remains unclear [3, 23]. Previous laboratory studies reported the ETP MIC at 4 μg/mL [3].

It is also important to further discuss the PK/PD of carbapenems when evaluating their efficacy. Carbapenems have a time-dependent mechanism of killing and exhibit a bacteriostatic effect when at least 20% of the exposure time is above the MIC. The bactericidal effect is achieved when the exposure time above the MIC is at least 40% [24]. A clinical study evaluating ETP pharmacokinetics in 12 patients with MDR or XDR TB found that the C_max_ was 127.5 μg/mL and the half-life was 2.4 h [25]. Considering that ETP requires only one daily dose, the blood concentration should remain above the theoretical MIC of 4 μg/mL for at least 8 h and additional administrations may be required if clinical isolates have a higher MIC. Similar data on FAR are still lacking, including studies on the early bactericidal activity, but some clinical trials are currently ongoing [26].

Stability is not a problem for FAR as it is thermo-stable at 37 degrees [27]. Ten clinical isolates (out of 20) showed different degrees of susceptibility to FAR and the addition of AMC further reduced the MIC in 6 clinical isolates. This is in line with previous experiments with other carbapenems, in particular MEM [8]. However, most of the MICs were very close to 8 μg/mL, which suggests we are reaching the limit of effectiveness. The current breakpoint for Gram positive bacteria is 2 μg/mL and 8 μg/mL for Gram negative microorganisms [28].

FAR did show some limited activity (MIC of 4 or higher) in strains completely resistant to MEM (MIC of 32 or higher). However, the five isolates fully susceptible to first line antituberculous drugs were completely resistant at the highest concentration of FAR used. This highlights a major issue of unpredictability and explains its limited use in clinical practice as no susceptibility pattern to traditional drugs can predict susceptibility to FAR and susceptibility testing is not available. In addition, further studies are still needed to assess which antibiotic level is actually achievable in the blood (and in the lung parenchyma) after the oral administration of FAR as reaching the concentrations tested in these experiments may be challenging.

Limitations of our study include the limited number of strains used, the analysis of in vitro data and the focus on the synergistic effect with AMC only. Other authors have also proposed the use of rifampin to provide additional synergy to carbapenems in the treatment of MTB and *M.abscessus* [29]. Further animal models or direct studies from patients with XDR strains may also be necessary to evaluate the real in vivo impact of complex treatment regimens including carbapenems on both the PK/PD of the drugs and their bactericidal effect.

The increasing global incidence of drug resistant TB demands additional therapeutic options. Although carbapenems are promising agents, this work has highlighted some of the limitations of their use. Information regarding clinical use and outcomes in humans is starting to emerge, showing results suggestive of activity against MTB. However, the contribution of the beta-lactam to the outcomes remains difficult to ascertain [26, 27]. Current opinions suggest that until more evidence becomes available, these drugs should be considered companion drugs rather than effective anti-TB agents [28], particularly in light of the administration route and higher cost associated to their use. Emergence of carbapenem resistance amongst gut microbiota is also an undesirable consequence of the use of these antibiotics and it is associated not only with the use of the drug but also with the duration of the exposure, that in the case of tuberculosis, is usually prolonged [30]. More evidence is needed to clarify the true impact of carbapenems in both TB treatment and outcome and as well as the financial burden, complications and microbiota ecological changes associated with their use to justify their re-classification as effective anti-TB agents.

## Conclusions

The lack of ETP activity may be associated with its degradation during incubation and a significant level of resistance was found in our experiments.

FAR may represent a more promising option as half of the isolates showed susceptibility and the addition of AMC further reduced the MIC in 7 isolates. However, most of the MICs were near the limit of effectiveness (8 μg/mL).

No susceptibility pattern to traditional drugs can predict susceptibility to FAR and susceptibility testing is not routinely available. PK/PD studies are needed as reaching the concentrations tested in these experiments may be challenging.

This work highlighted some of the limitations of carbapenem use. More evidence is needed to clarify their true impact in TB treatment and outcome, considering the financial burden, complications and microbiota changes associated with their use.

## Methods

### Selection of isolates

Twenty isolates in total (9 UK clinical isolates, 10 Russian clinical isolates plus H37Rv) were tested including drug susceptible, MDRTB and XDRTB. A full profile of the strains’ susceptibility, including meropenem, can be found in Table 2.

**Table 2:** List of *Mycobacterium tuberculosis* isolates tested against ertapenem and faropenem

Strain	Phenotypical resistance profile	Meropenem clavulanate MIC (μg/ml)	Notes
03:013	S	32	MonoR
03:039	H	16	MonoR
04:018	H, R, clari, ethi	Failed	MDR
05:094	Fully susceptible	8	
07:116	H, ethi	4	PolyR
11:136	S,H,R	> 32	MDR
11:156	S,H,R	4	MDR
11:191	H	16	MonoR
11:368	S,H,R	> 32	MDR
324	Fully susceptible	8	
333	S,H,R	2	MDR
346	S,H,R	2	MDR
347	Fully susceptible	> 32	
401	H,R	> 32	MDR
408	S, H, R	> 32	MDR
443	Fully susceptible	> 32	
548	N/A*	> 32	XDR
421	S, H, R, EMB, CAP, Moxi	> 32	XDR
433	S, H, R, EMB, PYR, CAP, Moxi	8	XDR
H37Rv	Fully susceptible	2	Control reference strain

*MDR* Multi-Drug resistant; *XDR* Extensively drug resistance; *MonoR* Resistant to one drug only; *PolyR* Resistant to more than one drug other than H/R. The resistance profile is shown in the second column, with a variety of fully susceptible, MonoR, MDR and XDR samples (*S* Streptomycin, *H* Isoniazid, *R* Rifampicin, *Clari* Clarithromycin, *Ethi* Ethionamide, *EMB* Ethambutol, *CAP*=Capreomycin, Moxi = Moxifloxacin, PYR = Pyrazinamide). The MIC against Meropenem/clavulanate is also shown

### Susceptibility testing

#### BACTEC960 MGIT system

Minimal inhibitory concentration testing was performed using the MGIT [18]. Four different concentrations of ETP and FAR were tested with and without the addition of AMC. These concentrations were selected based on previous pharmacokinetics/pharmacodynamics (PK/PD) data [12, 31]. All drugs were from Sigma-Aldrich (Dorset, UK) as either sodium (ertapenem/faropenem) or potassium (amoxicillin/clavulanate) salts and diluted in sterile water. The AMC potassium powder comes in a ratio of 2:1 and it was diluted to reach a final concentration of clavulanate of 2.5 μg/ml. A summary of the different concentrations used is provided in Table 3.

**Table 3:** Concentrations of ertapenem and faropenem tested

	Ertapenem	Ertapenem Clavulanate	Faropenem	Faropenem Clavulanate
	μg/mL	μg/mL	μg/mL	μg/mL
**BACTEC960 MGIT system**	16–8–4-2	16–8–4-2 (+ 2.5 Clavulanate each)	8–4–2-1	8–4–2-1 (+ 2.5 Clavulanate each)
**Microdilution**	0.25–0.5-1-2-4-8-16-32	0.25–0.5-1-2-4-8-16-32 (+ 2.5 Clavulanate each)	0.125–0.25-0.5-1-2-4-8-16	0.125–0.25-0.5-1-2-4-8-16 (+ 2.5 Clavulanate each)

Different concentrations of ETP and FAR were tested with and without the addition of AMC (2.5 μg/ml)

#### Microdilution

Microdilution testing was performed as previously described [21]. Briefly, 8 different concentrations of ERT and FAR were tested with and without the addition of AMC in microtiter plates, in Middlebrook 7H9 broth to a final volume of 100 μL. All the drugs were from Sigma-Aldrich (Dorset, UK). ERT and FAR were diluted in a mix of DMSO and distilled water and AMC were diluted in distilled water. A summary of the different concentrations used is provided in Table 3.

The concentrations tested were chosen based on previously published PK/PD data and the level of antibiotic achievable in the blood with the currently licensed dosing regimens [12, 31].

#### Controls

The reference strain H37Rv was used as a control strain. Growth controls without the addition of any drug were used in the MGIT testing as per manufacturer instructions. Positive (H37Rv without any drug) and negative (7H9 broth with no bacteria) controls were used in the microdilution method.

## Data Availability

Are available on request to the corresponding author TMcH.

## Abbreviations

MEM: Meropenem
ETP: Ertapenem
FAR: Faropenem
AMC: Amoxicillin-clavulanate
S: Streptomycin
H: Isoniazid
R: Rifampicin
Clari: Clarithromycin
Ethi: Ethionamide
EMB: Ethambutol
CAP: Capreomycin
Moxi: Moxifloxacin
PYR: Pyrazinamide
Tuberculosis: TB
MTB: *Mycobacterium tuberculosis*
MDR: Multi drug resistant
XDR: Extremely drug resistant
MIC: Minimum inhibitory concentration
MGIT: Mycobacterium growth incubator tube
PK/PD: Pharmacokinetics/pharmacodynamics
